# Myeloperoxidase: A New Biomarker of Inflammation in Ischemic Heart Disease and Acute Coronary Syndromes

**DOI:** 10.1155/2008/135625

**Published:** 2008-03-11

**Authors:** Valentina Loria, Ilaria Dato, Francesca Graziani, Luigi M. Biasucci

**Affiliations:** Institute of Cardiology, Catholic University of the Sacred Heart, 8 Largo Gemelli, 00168 Rome, Italy

## Abstract

Myeloperoxidase (MPO) is an enzyme stored in azurophilic granules of polymorphonuclear neutrophils and macrophages and released into extracellular fluid in the setting of inflammatory process. The observation that myeloperoxidase is involved in oxidative stress and inflammation has been a leading factor to study myeloperoxidase as a possible marker of plaque instability and a useful clinical tool in the evaluation of patients with coronary heart disease. The purpose of this review is to provide an overview of the pathophysiological, analytical, and clinical characteristics of MPO and to summarize the state of art about the possible clinical use of MPO as a marker for diagnosis and risk stratification of patients with acute coronary syndrome (ACS).

## 1. INTRODUCTION

Myeloperoxidase (MPO) is a well-known enzyme, mainly released by activated neutrophils, characterised by powerful pro-oxidative and proinflammatory properties. Recently, myeloperoxidase has been proposed as a useful risk marker and diagnostic tool in acute coronary syndromes and in patients admitted to emergency room for chest pain.

## 2. PATHOPHYSIOLOGICAL ROLE OF MYELOPEROXIDASE IN ISCHEMIC HEART DISEASE

Oxidative stress and inflammation play important roles in the pathogenesis of destabilization of coronary artery disease (CAD) leading to acute coronary syndromes (ACS). Infiltrating macrophages and neutrophils participate in the transformation of stable coronary artery plaques to unstable lesions [1, 2]. Recently, there has been a renewed interest in MPO, a proinflammatory enzyme that is abundant in ruptured plaque [3] and can be measured in peripheral blood. MPO is a hemoprotein that is stored in azurophilic granules of polymorphonuclear neutrophils and macrophages. MPO catalyzes the conversion of chloride and hydrogen peroxide to hypochlorite and is secreted during inflammatory condition. It has been implicated in the oxidation of lipids contained within LDL cholesterol. In addition, MPO consumes endothelial-derived NO, thereby reducing NO bioavailability and impairing its vasodilating and anti-inflammatory properties.

Major evidence for MPO as enzymatic catalyst for oxidative modification of lipoproteins in the artery wall has been suggested in a number of studies performed with low-density lipoprotein [4]. In contrast to low-density lipoprotein, plasma levels of high-density lipoprotein (HDL)-cholesterol and apoAI, the major apolipoprotein of HDL, inversely correlate with the risk of developing coronary artery disease. There is now strong evidence that HDL is a selective in vivo target for MPO-catalyzed oxidation, that may represent a specific molecular mechanism for converting the cardioprotective lipoprotein into a dysfunctional form, raising the possibility that the enzyme represents a potential therapeutic target for preventing vascular disease in humans [5]. Zhou et al. [6] showed that atorvastatin reduced serum MPO and CRP concentrations in patients with ACS.

MPO activity can be measured in blood and tissues by spectrophotometric assays using hydrogen peroxide and o-dianisidine dihydrochloride as substrates. In addition, MPO content can be measured in neutrophils as an index of degranulation with the Coulter counter and flow cytometry and circulating MPO by ELISA. Very recently, commercial methods allowing low-cost and high-volume measurements have been proposed. The introduction of these methods of measurement might make MPO a new and useful cardiac biomarker.

## 3. CLINICAL EVIDENCES FOR MPO AS A CARDIAC BIOMARKER

### 3.1. Primary prevention

There have been a few but important clinical studies examining the role of MPO as a marker of risk for CAD. Using an enzyme assay, Zhang et al. [7] showed that blood and leukocyte MPO activity were higher in patients with CAD than angiographically verified normal controls, and that this increased activity was significantly associated with presence of CAD (odds ratio, 11.9; 95% confidence interval (CI), 5.5–25.5). Results were independent of the patient's age, sex, hypertension, smoking, or diabetes status, LDL concentration, leukocyte count, and Framingham global risk score. More recently, Meuwese et al. [8], in the EPIC- (European prospective investigation into cancer and nutrition-) Norfolk prospective population study, have evaluated the association of MPO levels with the risk of future CAD in apparently healthy individuals. MPO was measured in baseline samples of a case-control study nested in the prospective EPIC-Norfolk population study: case subjects (*n* = 1138) were apparently healthy men and women who developed CAD during 8 years of follow-up; control subjects (*n* = 2237) matched for age, gender, and enrollment time, remained free of CAD. The MPO levels were significantly higher in case subjects than in control subjects and correlated with C-reactive protein (CRP) and white blood cell count. Risk of future CAD increased in consecutive quartiles of MPO concentration, with an odds ratio (OR) of 1.49 in the top versus bottom quartile. After adjustment for traditional risk factors, the OR in the top quartile remained significant at 1.36 (95% CI 1.07 to 1.73). Of interest in this study, serum MPO levels were associated with the risk of future development of CAD in apparently healthy individuals, but the association was weaker than that of traditional risk factors and CRP. However MPO, at variance from CRP, was largely independent from classical risk factors.

### 3.2. Secondary prevention

In ACS, MPO has been consistently found to be associated with the presence of instability and risk of future events in the studies that have explored these topics. Biasucci et al. [9] first observed that circulating neutrophils in patients with acute myocardial infarction (AMI) and unstable angina (UA) have a low MPO content, and therefore high MPO levels in the circulation, as compared with those with chronic stable angina and variant angina. This is indicative of a significant release of MPO from neutrophils related to their activation. The lack of neutrophil activation in patients with variant angina, and after stress test suggests that this phenomenon may occur independently of ischemic episodes. Therefore, MPO is prevalently a marker of instability and not simply a marker of oxidative stress and damage. Furthermore, in this study MPO did not correlate with CK-MB and troponin T release; this observation is clinically important as an extremely sensitive and specific marker of damage already exists (troponin), but no definite markers of instability exists so far. In this study, MPO content was determined on the Coulter counter, which measures the neutrophil count by flow cytometry and subsequently calculates the mean MPO content in that population.

Using the same method, Buffon et al. [10] studied 65 patients who underwent cardiac catheterization with coronary sinus sampling. The MPO content of the leukocytes collected from the arterial circulation and the coronary sinus effluent were compared. The authors found a gradient of MPO across the coronary circulation in patients with ACS and this gradient was present even when the culprit lesion involved with the ACS was in the distribution of the right coronary artery, which does not drain into the coronary sinus. In this study, as in the previous one, a significant correlation was found between systemic levels of C-reactive protein and either the aortic and coronary sinus neutrophil MPO.

**Figure 1:**
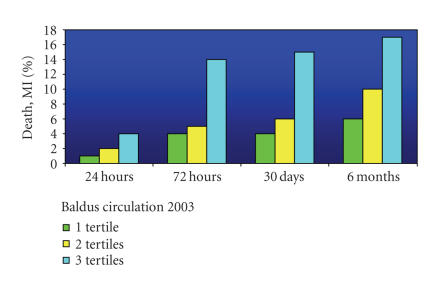
Increase in risk of death (D) plus MI (myocardial infarction) with increasing tertiles of MPO [11].

The potential usefulness for risk stratification of blood concentrations of MPO was examined in 2 recent studies. In the CAPTURE trial [11], MPO mass concentration was measured in 1090 patients with ACS. Rates of death and myocardial infarction (MI) were determined at 6months of follow-up. An MPO cutoff of 350 *μ*g/L was associated with an adjusted hazard ratio was 2.25 (95% CI, 1.32–3.82). The effects were particularly impressive in patients with undetectable cardiac troponin T (cTnT < 0.01 *μ*g/L), in whom the hazard ratio was 7.48 (95% CI, 1.98–28.29). Of interest, the increase in risk was already evident after 72 hours, increasing only slightly thereafter (Figure 1). This observation is in keeping with the data by Biasucci et al. [9] who had shown return of MPO to baseline levels in all patients, including those with myocardial infarction, within one week. This point is important, as suggests a peculiar characteristic of MPO, at variance from other inflammatory markers commonly used (as CRP, fibrinogen) and from other proposed inflammatory markers that remain elevated for relatively long time or have an extremely short and unreliable half-life (such as interleukins). The predictive value of MPO was independent by C-reactive protein and high MPO serum levels indicated increased cardiac risk both in patients with medium C-reactive protein serum levels (20.0% versus 5.9%; *P* < .001) and in those with low C-reactive protein serum levels (17.8% versus 0%; *P* < .001), suggesting that recruitment and degranulation of neutrophils is a primary event and is followed by release of other systemic mediators and acute-phase proteins such as C-reactive protein. At variance from CRP levels, levels of MPO were not influenced by troponin, suggesting a prognostic role of MPO independent from troponin and confirming that inflammation is a primary phenomenon in ACS.

In a study of 604 consecutive patients presenting to the emergency department with chest pain, Brennan et al. [12] demonstrated a progressive increase in odds ratios for major adverse events at 30 days and 6 months with each quartile increase in MPO concentration in patients with negative troponin. The 6-month outcomes were similar to the results of the CAPTURE trial: corresponding odds ratios were 1.6 (95% CI, 1.0–2.7), 3.6 (2.2–5.8), and 4.7 (2.9–7.7) for the second, third, and fourth quartiles, respectively (cut offs of 119, 198, and 394 pmol/L, resp.). It is of interest that, even in the absence of myocardial necrosis and in negative cardiac troponin patients, baseline measurements of MPO significantly enhanced the identification of patients at risk, although this study included the “need for revascularization” within the definition of major adverse cardiac events and used an inappropriately high cTnT cutoff of 0.10 *μ*g/L. Furthermore, while troponin T takes three to six hours to rise to measurable circulating levels after myocardial injury, MPO levels were significantly elevated at baseline (even within two hours after the onset of symptoms) in patients who were initially negative for troponin T. These findings suggest that measurement of MPO levels may be useful in triage in the emergency department and that elevated plasma MPO levels may be a marker of unstable angina preceding myocardial necrosis and therefore, a predictor of vulnerable plaque. Interestingly, C-reactive protein levels predicted the risk of myocardial infarction at presentation for the entire cohort but were not predictive of major adverse cardiac events in the group that was negative for troponin T. In the cohort that was consistently negative for troponin T, the areas under the curve for MPO were significantly higher than those for troponin T, CK-MB, and C-reactive protein.

Taken together, these data suggest that CRP and MPO may be complementary and explore different fields: CRP is a marker of disease activity and vascular inflammation, and is useful for long-term risk stratification while MPO is a marker of plaque instability and neutrophil activation and may be associated with short-term stratification, in particular in patients with troponin negative levels.

More recently, several studies have also investigated the value of MPO in predicting long-term outcomes. Recently, S.-H. Li et al. [13] have studied 176 consecutive patients who underwent coronary angiography. The patients were divided into four groups according to the quartile of MPO level. They have found that: (1) ACS rate (36.2%) in the fourth quartile group of MPO level was 6 times higher than that in the first quartile group of MPO level, *P* < .01. (2) Gensini score in the fourth quartile group of MPO level was significantly higher than that in the first quartile group (*P* < .01). WBC in the fourth quartile group was also significantly higher than that in the first quartile group, *P* < .05. In addition, Kaplan-Meier event rate curve showed that there was a significant difference in outcome (death, AMI, revascularization) between the groups with MPO ≥ 62.9 AUU/L and ≤62.9 AUU/L of MPO serum level at 6-month follow-up visit (chi(2) = 13.5, *P* = .01). Furthermore, Cavusoglu et al. [14] have investigated the long-term prognostic significance of baseline MPO levels in a well-characterized cohort of 193 men with ACS. All patients were followed prospectively for the development of death and MI, and follow-up data were available for all patients at 24 months. Using the median MPO value of the entire cohort of patients (20.34 ng/mL) as a prespecified cutoff, the MI-free survival at 24 months for the group with MPO values below cutoff were significantly lower than in those with values above cutoff.

Mocatta et al. have investigated the relationship between plasma MPO and clinical outcome after AMI [15, 16]. They have studied 512 AMI patients at hospital admission and have measured plasma MPO in AMI patients and found a significant association of MPO with follow-up events. Importantly, MPO was of incremental prognostic value on the top of ejection fraction and BNP, a finding observed also by Khan in a similar population of patients with STEMI [17].

## 4. CONCLUSIONS

MPO is a marker of inflammation and oxidative stress that has been consistently demonstrated to be elevated in patients with ACS. However, the data so far available are relatively few; therefore, more studies are requested to precisely define the role of MPO. In particular all studies involving MPO assessment have used different methods, thus a standardization effort is needed. Furthermore, increased MPO is not likely to be specific for cardiac diseases, as activation of neutrophils and macrophages can occur in any infectious, inflammatory, or infiltrative process, therefore, more studies should address and clarify these points. So far, as reported in Table 1, MPO seems to have, among the inflammatory markers, a role superior to that of pregnancy-associated plasma protein-A (PAPP-A), CD40 ligand (CD40L), and cytokines, but still inferior to CRP.

## Figures and Tables

**Table 1:** Prognostic and diagnostic value and analytical performance of inflammatory markers in CAD: myeloperoxidase (MPO), C reactive protein (CRP), pregnancy-associated plasma protein-A (PAPP-A), CD40 ligand (CD40L), and interleukins.

Inflammatory markers in CAD			
	Prognosis	Diagnosis	Analyt. perform.
MPO	+++	++	++ (more?)
CRP	++++	+/−	++++
PAPP-A	++	+	−
CD40L	++	+/−	−
Interleukins	+++	+/−	−
